# The Seven Books of Paulus Ægineta. Translated from the Greek. With a Commentary Embracing a Complete View of the Knowledge Possessed by the Greeks, Romans, and Arabians on All Subjects Connected with Medicine and Surgery

**Published:** 1845-07

**Authors:** 


Art. VI.—1. *The Seven Books of Paulus Ægineta. Translated from the Greek. With a Commentary embracing a complete view of the knowledge possessed by the Greeks, Romans, and Arabians on all subjects connected with Medicine and Surgery.* By Francis Adams. In Three Volumes. Vol. I.—*London, Printed for the Sydenham Society,* 1844. 8vo, pp. 684.

2. *Observations on Aneurism, selected from the Works of the principal writers on that Disease from the earliest periods to the close of the last century.* Translated and Edited by John E. Erichsen, Lecturer on General Anatomy and Physiology at the Westminster Hospital.—*London, Printed for the Sydenham Society,* 1845. 8vo, pp. 524.

3. *Animal Chemistry, with reference to the Physiology and Pathology of Man.* By Dr. J. Franz Simon, Fellow of the Society for the Advancement of Physiological Chemistry at Berlin, &c. &c. Translated and Edited by George E. Day, m.a. & l.m. Cantab., Licentiate of the Royal College of Physicians. In Two Volumes. Vol. I.—*London, Printed for the Sydenham Society,* 1845. 8vo, pp. 360.

Our readers are aware of our determination not to *review* any of the works of this society; and the excellent choice of works hitherto made by the Council, and the admirable execution of their important and difficult tasks by the respective editors, give us no opportunity to *criticise* them. It appears from the Treasurer's Report, that the number of subscribers who contributed to the funds of the Society last year, was 2032; and while the Society can afford to supply each member, in return for his guinea, with three such splendid volumes as are now before us (the selling price of which in the shops, at the ordinary rate, would be at least *double* the subscription,) we shall have, every Quarter, less and less reason for wishing to convey to the profession information respecting these publications. A few years more, and every member of the profession who studies his science and his art, must be a member of the Sydenham Society.

We cannot better or more concisely express our sentiments as to the value of the first two works on our list, than by quoting the words of the learned and excellent secretary, Dr. Bennett, as given in the Third Report of the Society just published.

"If among the original objects of the Sydenham Society those which relate to ancient Medical literature are to be attained at all, the Council feel satisfied that in the estimation of all whose opinion is of value, these objects are likely to be gained by the publication of such a work as the 'Paulus Ægineta' of Mr. Adams. Replete with learning, and comprising the most complete view that has ever been given of the knowledge possessed by the Greeks, Romans, and Arabians, on all subjects connected with medicine and surgery, this work will prove a lasting monument of the industry and erudition of the editor, and an honour to his country. ....

"The direct practical importance of an acquaintance with the labours of our forefathers is well illustrated by the volume of 'Observations on Aneurism,' in which are contained, amidst much that will be interesting and valuable to the surgeon, the results of former trials of certain plans of treatment, which, after having fallen into desuetude, are, in the present day, again claiming attention. The Council have the satisfaction of knowing that, in the estimation of the highest existing authorities on this subject, a valuable and timely boon has been conferred on the profession by the publication of the 'Observations on Aneurism.' (pp. 3-4.)

Of the third work, we need say nothing more in commendation than that it is worthy to take rank with its fellows. It was deprived of the benefit of Dr. Bennett's verdict, simply because it was not printed at the date of his Report.

We must give one word of commendation to the Council of the Society, for their attention to the minor, but still not unimportant qualities of their publications. The style in which the volumes are got up is very superior. The paper and print are excellent, and the binding chaste and elegant.

